# Modelling and Manufacturing of a 3D Printed Trachea for Cricothyroidotomy Simulation

**DOI:** 10.7759/cureus.1575

**Published:** 2017-08-18

**Authors:** Gregory Doucet, Stephen Ryan, Michael Bartellas, Michael Parsons, Adam Dubrowski, Tia Renouf

**Affiliations:** 1 Faculty of Engineering and Applied Science, Memorial University of Newfoundland; 2 Faculty of Medicine, Memorial University of Newfoundland; 3 Medicine, Memorial University of Newfoundland; 4 Emergency Medicine, Memorial University of Newfoundland; 5 Emergency Medicine, Pediatrics, Memorial University of Newfoundland

**Keywords:** 3d printing, medical simulation, emergency medicine, cricothyroidotomy, simulation

## Abstract

Cricothyroidotomy is a life-saving medical procedure that allows for tracheal intubation. Most current cricothyroidotomy simulation models are either expensive or not anatomically accurate and provide the learner with an unrealistic simulation experience. The goal of this project is to improve current simulation techniques by utilizing rapid prototyping using 3D printing technology and expert opinions to develop inexpensive and anatomically accurate trachea simulators. In doing so, emergency cricothyroidotomy simulation can be made accessible, accurate, cost-effective and reproducible.

Three-dimensional modelling software was used in conjunction with a desktop three-dimensional (3D) printer to design and manufacture an anatomically accurate model of the cartilage within the trachea (thyroid cartilage, cricoid cartilage, and the tracheal rings). The initial design was based on dimensions found in studies of tracheal anatomical configuration. This ensured that the landmarking necessary for emergency cricothyroidotomies was designed appropriately. Several revisions of the original model were made based on informal opinion from medical professionals to establish appropriate anatomical accuracy of the model for use in rural/remote cricothyroidotomy simulation.

Using an entry-level desktop 3D printer, a low cost tracheal model was successfully designed that can be printed in less than three hours for only $1.70 Canadian dollars (CAD). Due to its anatomical accuracy, flexibility and durability, this model is great for use in emergency medicine simulation training. Additionally, the model can be assembled in conjunction with a membrane to simulate tracheal ligaments. Skin has been simulated as well to enhance the realism of the model. The result is an accurate simulation that will provide users with an anatomically correct model to practice important skills used in emergency airway surgery, specifically landmarking, incision and intubation. This design is a novel and easy to manufacture and reproduce, high fidelity trachea model that can be used by educators with limited resources.

## Introduction

Cricothyroidotomy is an emergency medicine procedure that allows for tracheal intubation. Cricothyroidotomies are necessary to establish ventilation in life-threatening situations such as foreign body airway obstruction, extensive facial trauma, or angioedema. The procedure involves making a mid-line incision through the skin and cricothyroid membrane, creating a patency that allows oxygenation [1]. It is generally used as a last resort if oxygenation and ventilation cannot be established. The procedure, while invasive, has few complications and can save lives in emergencies [2]. For this reason, it is an important procedure for front-line care providers responsible for airway management to learn. Simulation is an effective approach to build and maintain competency in this procedure.

Simulation-based medical education (SBME) is an approach that focuses on practice, error-correction, and debriefing, which ultimately has a positive effect on patient safety [3]. It enables the teaching of technical skills across many medical disciplines, which has incited enthusiasm within the medical community. Thus, the field of simulation is evolving rapidly [4].

The increased demand for high quality, cost-effective medical simulation has led to the incorporation of three-dimensional (3D) printing into the field, and it is now changing the approach to SBME. Computer-aided design (CAD) allows one to create novel anatomical models at a low cost, and this has increased the accessibility of simulation technologies [5-6]. The integration of novel simulation tools allows teachers to overcome barriers such as rare pathology and low patient volumes in developing a competency-based medical education environment. This has been suggested in many areas of simulation research, such as ultrasound guidance [7], airway management [8], and lumbar puncture [9]. This paper discusses the development and production processes of a novel 3D printed trachea for simulation-based cricothyroidotomy education.

The quality of a simulation model is generally judged based on how well it matches the appearance and/or the behavior of the simulated anatomy and/or system - known as fidelity [10]. Engineering fidelity describes the physical characteristics of the model and its effectiveness in mimicking anatomical attributes [11]. As such, mentions of fidelity in this technical report will refer to engineering fidelity.

**Figure 1:**
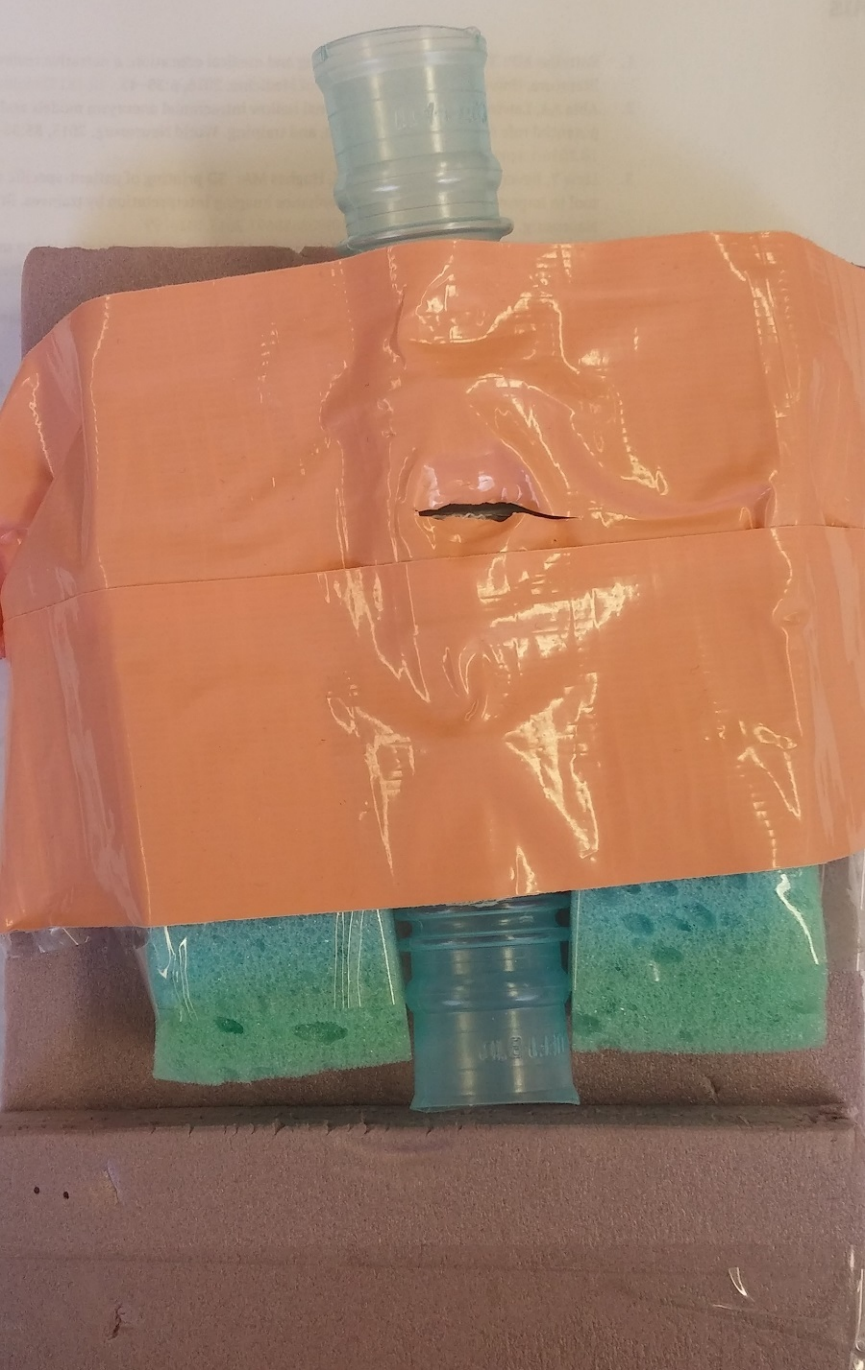
Currently used rural cricothyroidotomy simulator

Currently, the low fidelity models being used in low resource settings around the world, including in Newfoundland and Labrador, are composed of low-cost everyday objects which mimic human anatomy for procedural practice (Figure 1) [12]. However, these low-cost solutions often sacrifice engineering fidelity. By contrast, the model developed for this technical report is also a low-cost solution, in that it is inexpensive, durable, reusable, and reproducible, but it is also anatomically correct. Through early engagement with end-users and revisions of the design (rapid prototyping), the goal is to be able to produce a cricothyroidotomy training tool in a cost-effective manner.

## Technical report

Protocol

We followed a process proposed by Cristanchio et al. termed "Aim – FineTune – Follow Through", rooted in frameworks from psychology, motor learning, education, and experimental design to enable initial design and expert opinion-based rapid prototyping [13]. Specifically, the initial prototype was developed based on input from a single emergency medicine doctor. Next, the prototype was printed and, to further refine the anatomical accuracy of the model, the opinions of medical experts were sought. These opinions were used to gauge the anatomical accuracy of each iteration of the model and to identify any possible areas of improvement. When considering the effectiveness of each model, it was essential that the experts not only considered the anatomical accuracy of the model but also how it would fit mechanically into the simulation scenarios. This iterative approach, while informal, proved to be effective, as the model improved significantly from the initial design to the final model. A graphic representation of the review process is shown in Appendix A.

3D printing equipment

There were two fused deposition modelling (FDM)-type printers used to create the multiple iterations of the trachea model, the Lulzbot Taz 6 (Loveland, Colorado, USA) and the Ultimaker 2+ (Geldermalsen, Netherlands). These were chosen because of their printing speed and high print resolution, which leads to quick, high quality simulation models.

Although choosing a 3D printer is crucial, selecting the appropriate filament (printing material) to best suit your needs can be even more important. The most commonly used filaments are polylactic acid (PLA) and acrylonitrile butadiene styrene (ABS) plastics; both filaments are hard and rather brittle once printed. On the other hand, filaments such as SemiFlex by NinjaTek (Manheim, Pennsylvania, USA) are flexible when printed. This is desirable when mimicking the flexibility of the trachea. Three different filaments were used during this project, InkSmith PLA-I Filament (Chicago, Illinois, USA); M3D FLX (Bethesda, Maryland, USA); and SemiFlex by NinjaTek (Manheim, Pennsylvania, USA).

Printing parameters

The software interface used to print the trachea model was the Lulzbot Taz version of CURA 21.00 (Aleph Objects, Colorado, USA). This interface allows the user to change printing parameters and preview the model in the software before printing begins. Before changing any printing parameters, the user must develop a sound understanding of the factors involved in printing. A brief overview of the printing parameters is provided in Appendix B.

Key elements of cricothyroidotomy simulation

**Figure 2:**
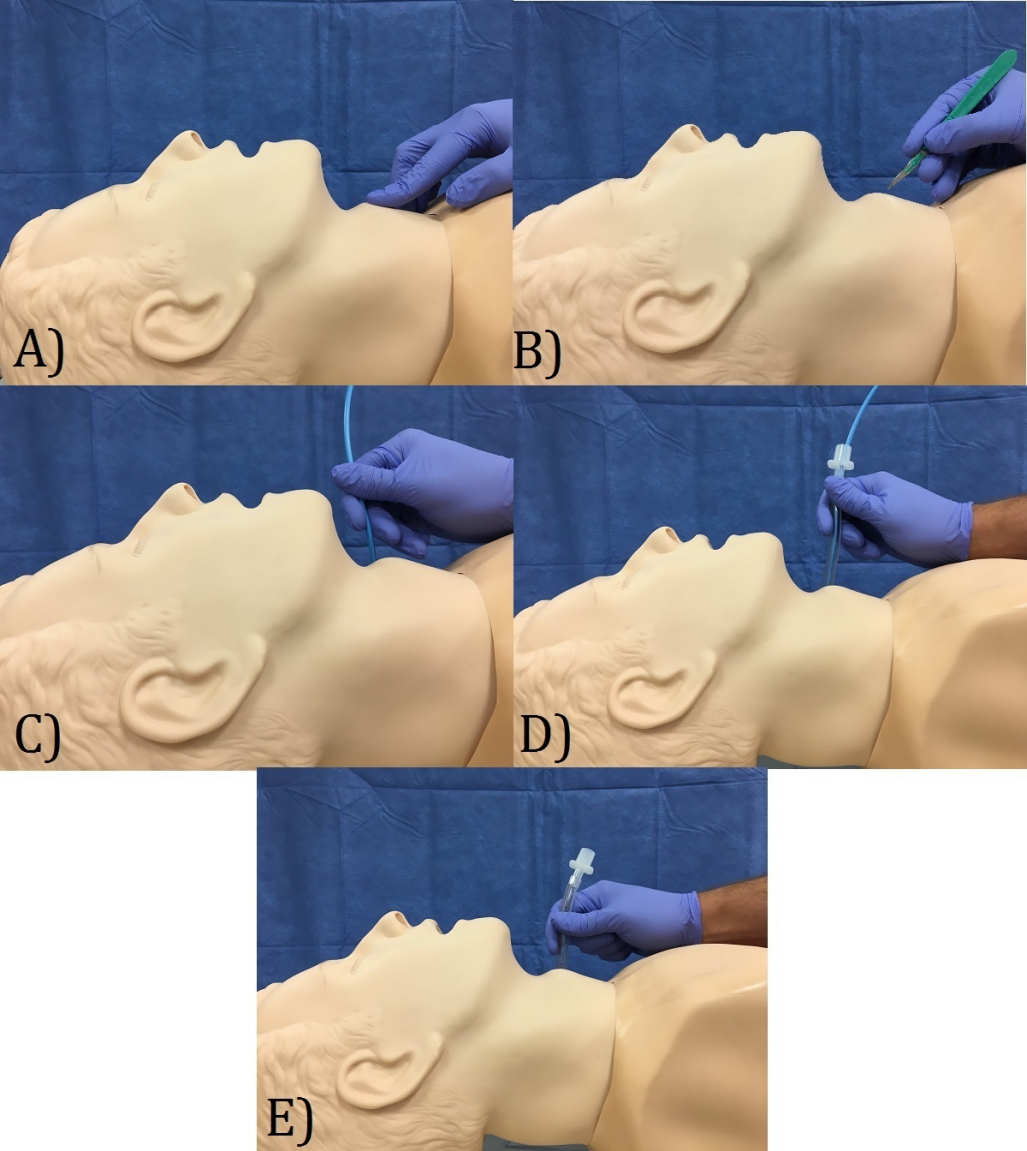
Surgical cricothyroidotomy five step technique: a) palpate the cricothyroid gap; b) create small 1.5 cm incision; c) insert bougie to confirm tracheal intubation; d) insert intubation tube; e) allow for oxygenation

There are several features of the cricothyroidotomy simulation model that make it a realistic simulation [14]. If the general shape, length, and width of the trachea are anatomically accurate, the model will be sufficient for simulation purposes. The most important parts of the model to consider are the thyroid and cricoid cartilages. These two segments are essential landmarks for incision. An effective model will allow users to distinguish between the tracheal rings and cricoid cartilage and accurately determine the position of the gap between the thyroid and cricoid cartilages. The model must also allow the user to confirm that tracheal, as opposed to esophageal, intubation has been established. The essential criteria outlined were deemed crucial to the simulation after researching the technique [15] and consulting with medical experts within the Department of Emergency Medicine at Memorial University, Newfoundland (MUN) (Dr. Michael Parsons, Dr. Adam Dubrowski, and Dr. Tia Renouf were the experts consulted) [A1]. A visual aid outlining the cricothyroidotomy can be found below (Figure 2). 

Techniques used in research and design of the trachea

Several dimensions and geometric relations are considered critical to the overall design, including the general shape, cross-sectional length, width, and height of the cricoid and thyroid cartilages and tracheal rings. The overall length of the trachea was also considered an essential dimension. Literature describing the exact measurements of the trachea were referenced to create the first iteration of the design [16-18] and is summarized in Appendix C. Interpretation of the literature led to the identification of crucial design criteria shown below in Table 1.

**Table 1:** Design requirements of major sections of the tracheal model

Specific Anatomy	Area of Focus
Cross-sectional area of trachea	For anatomical accuracy, the generic circular shape of the trachea was reproduced with one slight modification. The bottom of the trachea was flattened so the model could be fixed onto a surface during simulation (Figure 3.1). The size of the trachea needed to fit the ET (endotracheal) tube (11.2 mm diameter) and provide space for it to move around, similar to a real trachea.
Longitudinal area of trachea	Not only should the total length of the trachea be anatomically accurate, but the tracheal rings must be replicated. The tracheal rings must be spaced correctly for realism during the passage of the bougie.
Thyroid cartilage	The thyroid cartilage is crucial for landmarking. As such, the shape and size of the protrusion must provide realistic feedback to the user during landmarking. This feedback provided by the model during landmarking is controlled by adjusting the angle and length of the thyroid cartilage’s protrusion.
Cricoid cartilage	Landmarking of the correct incision point is provided by both the thyroid and cricoid cartilages. The cricoid cartilage is thicker than the tracheal rings and must also protrude past them to be distinguishable for landmarking.
Incision point	The success of the entire simulation scenario depends the ability for a proper incision to be made. Adjusting the gap between the thyroid and cricoid cartilages allows for the realism of the incision point to be mimicked. The diameter of the ET tube (11.2 mm) played a role in determining the size of the gap. The size of this gap could be controlled by adding space between the cricoid and thyroid cartilages, but also but changing the geometries of the aforementioned cartilages.

Results

Adhering to the process proposed by Cristanchio et al. termed "Aim – Fine Tune – Follow Through" [13], we engaged in a number of cycles of the streamlined design, manufacturing, and revision processes (Appendix A). Consequently, we were able to develop an effective 3D printed tracheal model at a low cost. The average material cost of all of the models (Figure 3.1) was $2.85 CAD and the cost of the final model was $3.63 CAD. This cost excludes the cost of saran wrap which was used to mimic the tracheal ligaments and the artificial skin (Figure 3.2) which together would cost $5.00 CAD.

**Figure 3:**
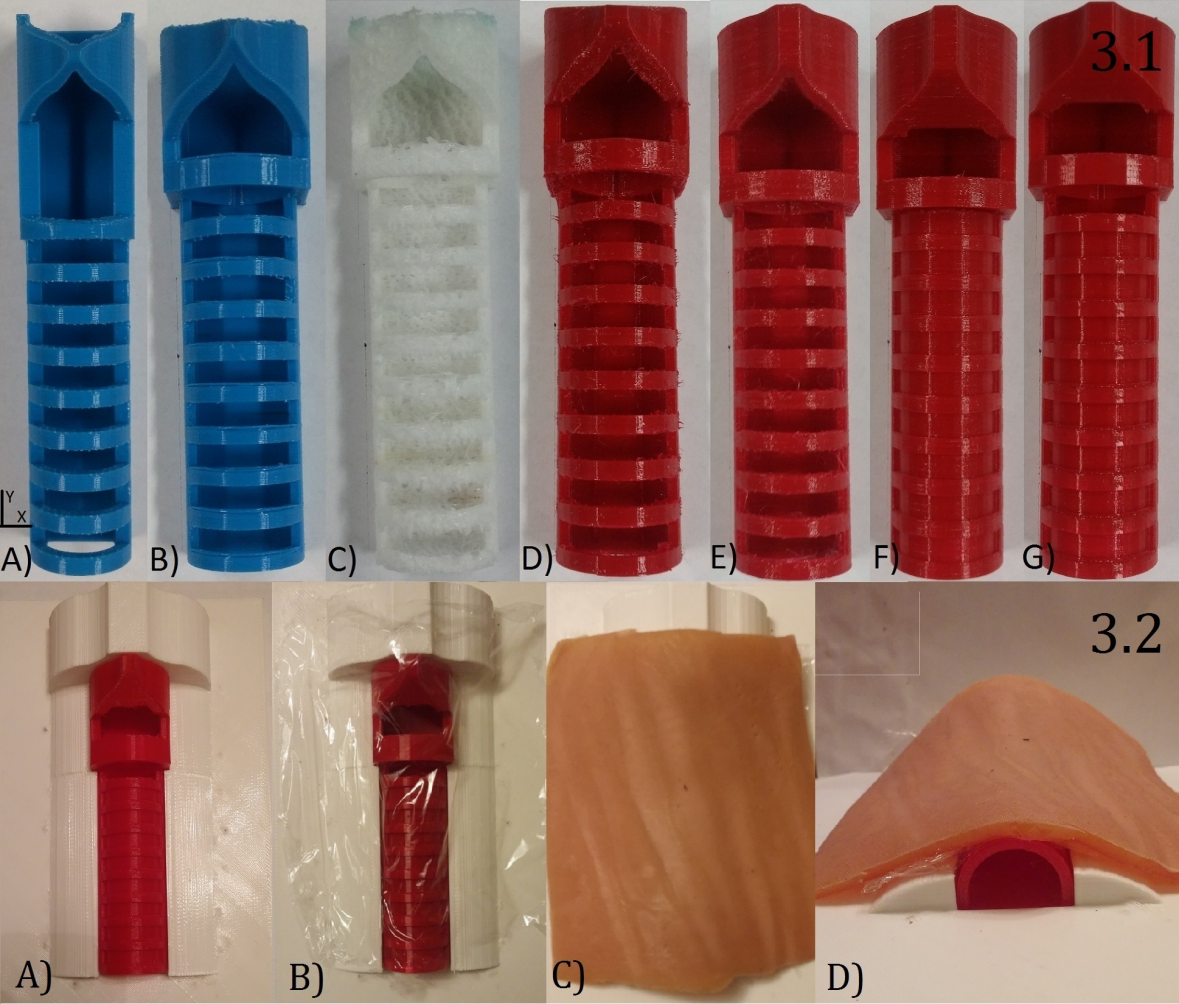
3.1 Front view of tracheal model iterations: a) initial design; b) revision 1; c) revision 2; d) revision 3; e) revision 4; f) revision 5; g) final design; 3.2 a) trachea model mounted in custom base; b) trachea model covered with saran wrap to simulate the tracheal ligaments; c) top view of trachea covered with artificial skin; d) front view of trachea model covered with artificial skin

The final product was printed on the Lulzbot Taz 6 using SemiFlex filament. The printer used for the final model costs $3429.99 CAD and the roll of filament used for the final design costs approximately $80.00 CAD for a 0.75 kg roll (22 models).

The average manufacturing time of the models was three hours and 58 minutes and the print time of the final model was three hours and 16 minutes. The quick print time and ability to make design changes easily makes the model great for simulation. By using SolidWorks (Waltham, Massachusetts, USA) to design the model, quick changes could be made to the design. This allowed medical experts and engineers to collaborate and produce an anatomically accurate model. Because of the quick manufacturing time and the ease of manufacturing, it allowed for each iteration to be 3D printed. This made the informal review of each iteration possible and allowed for an effective design refinement process.

To complete the informal review, the pre-determined crucial criteria (Table 1) were used. These aspects of the simulation were considered throughout the design process and were the areas of focus during the revision phase. A description of the initial dimensions and how they were used as well as the results of the subjective assessment of each revision were recorded (Appendix C). Although the informal review of the model by medical professionals was essential to its refinement, there were also 3D printing-related constraints to consider with each iteration. The most prominent of these constraints was the structural integrity of the model.

We considered the structural integrity of the design from two different perspectives. Firstly, the model must be able to withstand the forces applied to it during the simulation itself, but the structural integrity could be compromised in a completely different way during printing itself. As such, printing parameters (Appendix D) and print orientation played major roles in the manufacturing process. It should be noted that all models were printed with the top down (opposite of Figure 3) to ensure structural integrity and to reduce any excess scaffolding. Scaffolding is the support material used by 3D printers to provide a printing platform for any overhanging features of the print.

All previously discussed dimensions and geometries were the foci of the model review as they, if controlled properly, would provide an accurate simulation model. The iterative analysis led to the successful creation of an easily reproducible design.

## Discussion

The final iteration of the tracheal design met all the criteria required to properly simulate a cricothyroidotomy. The model enables potential learners to properly landmark the incision point, create a proper incision, and use the bougie effectively; it also allows for realistic intubation to be achieved. In addition, the models were developed as cost-effective, easily reproducible solutions.

Although a high-end desktop printer was used to create the final revision, the tracheal model was designed to be printer-friendly and reduce the chance of print failure. To do so, all unnecessary overhangs in the model were eliminated and the print orientation ensured that the base of the model was the largest part of the model, ensuring structural integrity during printing. The flat base allows for the model to build on itself as opposed to building on scaffolding, making the print sturdy from start to finish. Eliminating the need for excess scaffolding also reduces the cost of printing the model, making the simulator even more efficient. Determining the optimal balance between anatomical accuracy and 3D printability is difficult but critical when designing a realistic simulation model.

The accuracy of the landmarking on the tracheal model makes it great for emergency cricothyroidotomy simulations. The model can be used concurrently with a thin membrane to simulate the tracheal membrane and artificial skin, to complete the realistic, high fidelity simulator (Figure 3.2). 

Low-cost simulation models are advantageous in medical education as they allow financially restricted medical institutions to provide their students and staff with realistic simulation scenarios. This type of simulation is also ideal for remote/rural medical establishments as it reduces shipping time and expenses by allowing for in-house manufacturing. The tracheal model has been made open source to provide the aforementioned institutions access to this particular simulation model. Teachers and learners who wish to use the final design of the model can acquire the .STL file by contacting the authors or use the engineering drawings provided (Appendix D).

## Conclusions

This process has shown that the combination of quantitative research and opinion-based review by experts leads to a streamlined design process and makes it relatively easy to create an anatomically accurate simulation model. The rapid evolution of 3D printing technology makes it an attractive option for manufacturing anatomical models.

Cost-effectiveness and rapid prototyping are the two major advantages of 3D printing. Furthermore, the technology offers the ability to prototype patient-specific models/rare pathologies for use in patient education and medical simulation. In the future, one area that could be improved upon is the realism of the 3D printing materials when compared to biomaterials. By adjusting the printing parameters and filament types used, one can completely mimic the exact feel and mechanical properties of biomaterials. Once there is a clearly defined method to thoroughly mimic biomaterials such as ligaments and skin, even more realistic anatomical models can be produced, thereby enhancing the field of medical simulation.

## Figures and Tables

**Figure 4:**
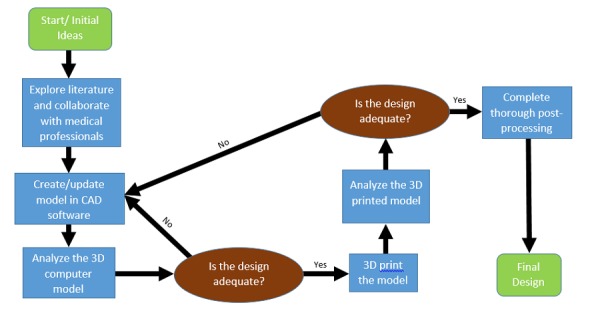
Design methodology flow chart

**Figure 5:**
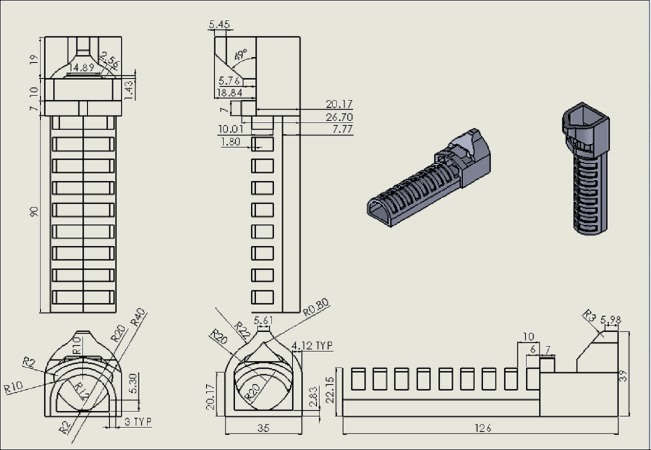
Engineering drawings of final tracheal model

## Appendices

Appendix A: Design methodology

Appendix B: Printing parameters

**Table 2:** A brief guide to printing parameters

Parameter Type	Description
Quality	The definition of quality varies from print to print. If the print requires high resolution, *Layer Height* is the most important factor to consider whereas *Shell Thickness *controls the overall strength of the model. *Shell Thickness *controls the thickness of the print’s side walls.
Fill	Although *Shell Thickness* is the main factor that determines the print strength, to create an evenly strong print, the *Bottom/Top Thickness* must also be considered. Not only does the *Fill Density *affect the strength of the model, but applying fill density will supply internal support for the top layers to be printed on.
Speed and temperature	*Print Speed *not only controls the overall print time, but can have a major effect on the overall print quality and even the success of the print. Both *Printing Temperature *and *Bed Temperature* are parameters that determine the success of the print. If these two factors are not properly controlled, it can cause issues with extrusion and bed adhesion.
Support	It is not required for every print, but selecting an appropriate *Support Type *can solely determine the success of a print. Support structures are used as scaffolding to provide a surface to print on for overhanging parts of a print. Depending on the geometry of the print, a type of *Platform Adhesion* (brim or raft) may be needed to provide a solid base for the print to build on.
Filament	Inputting the correct filament *Diameter* is necessary for proper extrusion to occur. *Flow% *is usually increased when printing with flexible materials that are difficult to extrude.
Advanced	These settings typically remain unchanged but advanced users can adjust them based on the specific requirements of a print.

Appendix C: Initial design and iterative changes

*Initial Design*: Dimensions A-B, C-D, T-U, and V-X [14] that Randestad provides were used as guides for creating the cross sectional area of the cricoid cartilage and the tracheal rings. The sagittal height and width were taken from dimensions L-M and L-L [14]. The thickness of the cricoid cartilage was determined by taking the average of both the mean value from men and women [15]. The overall tracheal length was taken from Table 5 [15]. 

To model the thyroid cartilage, dimensions 1, 16, and 3 [16] were used to represent height, width, and length respectively. The obtuse angle of the protrusion from the sagittal view was modelled based on the general shape shown in Figure 1 (D) [16]. The distance between the cricoid and thyroid cartilages was gathered from dimension 18 [16].

To better suit the needs of the simulation, the backside of the tracheal model was flattened so that the model could be set down without it rolling away. The model was made out of PLA-I with specific printing parameters (Table 3).

**Table 3:** Iterative design revision and analysis

Iteration	Modifications/Revisions	Analysis
1	N/A	Gap between thyroid and cricoid cartilage was too large in the y-direction Cricoid cartilage should be easily distinguishable from the tracheal rings Angle of protrusion of the thyroid cartilage was too sharp
2	Gap between thyroid and cricoid cartilages was shortened Cricoid cartilage was raised from the tracheal rings Angle of protrusion was flattened slightly Distance of protrusion of the thyroid cartilage was increased	Model didn’t mimic the flexibility of the tracheal rings
3	Same design as iteration 2 used but FLX filament was used	Material was much too pliable The angle on the top of the thyroid cartilage was too steep
	SemiFlex filament was used to mimic flexibility while maintaining strength The angle on top of the thyroid cartilage was decreased slightly	The angle on top of the thyroid cartilage caused there to be excess scaffolding and in turn unnecessary surface roughness
5	The top of the thyroid cartilage was flattened	The space between the tracheal rings introduces potential for the bougie to get caught The bottom of the thyroid cartilage isn’t a ridge; rather, it is more similar to a platform
6	A thin wall was inserted concentrically with the existing tracheal rings to prevent the bougie getting stuck A thin plane was added between the walls on the bottom of the thyroid cartilage	The tracheal rings were now much less pronounced due to the addition of the concentric wall The thin wall between the cricoid cartilage and first tracheal ring caused the bougie to get caught The flat surface on top of the thyroid cartilage created a large platform that was unrealistic
7	The tracheal rings were raised to make them more prominent on both the inside and outside of the trachea Between the cricoid cartilage and first tracheal ring, the thin concentric wall was removed The flat surface created by the thyroid cartilage was shortened to make it more of a point rather than a platform	N/A

Appendix D: Guidelines to recreation of model

 By using the dimensions provided above, in conjunction with the recommended parameters provided below in Table 4, the model used in this simulation can be easily replicated.

**Table 4:** Recommended print settings

Parameter	Value
Layer height	0.25 mm
Wall thickness	1 mm
Fill density	20%
Nozzle size	0.4 mm
Print speed	50 mm/s
Print temperature	230 deg C
Print bed temperature	70 deg C
Bottom Layer Speed	20 mm/s
